# Fractures of implant fixtures: a retrospective clinical study

**DOI:** 10.1186/s40902-020-00258-3

**Published:** 2020-04-25

**Authors:** Han-Chang Yu, Young-Kyun Kim

**Affiliations:** 1 Department of Oral and Maxillofacial Surgery, Section of Dentistry, Seoul National University Bundang Hospital, 82 Gumi-ro 173, 173beon-gil, Bundang-gu, Seongnam, 13620 Korea; 2 Department of Dentistry and Dental Research Institute, School of Dentistry, Seoul National University, Seoul, South Korea

**Keywords:** Dental implant, Implant fracture

## Abstract

**Background:**

The aim of this study was to evaluate the factors that may affect implant fixture fractures.

**Methods:**

Patients who experienced implant fixture removal at Seoul National University Bundang Hospital from 2007 to 2015 due to implant fixture fracture were included. Implant/crown ratio, time of implant fracture, clinical symptoms before implant fracture, treatment of fractured implants, and the success and survival rate of the replaced implants were evaluated retrospectively.

**Results:**

Thirteen implants were fractured in 12 patients. Patient mean age at the time of fracture was 59.3 years. Of the 13 implants, 7 implants were placed at our hospital, and 6 were placed at a local clinic. The mean crown/implant ratio was 0.83:1. The clinical symptoms before fracture were screw loosening in five implants, marginal bone loss in five implants, and the presence of peri-implant diseases in five implants. All the fractured implants were removed, and 12 out of the 13 sites were re-implanted. Parafunctions were observed in two patients: one with bruxism and one with attrition due to a strong chewing habit.

**Conclusions:**

Several clinical symptoms before the fracture of an implant can predict implant fixture failure. Therefore, if these clinical symptoms are observed, appropriate treatments can be taken before more serious complications result.

## Background

Mechanical complications are an issue in implant restorations. Among the complications, implant fixture fractures rarely occur, but when they do, they should be re-installed after removal, or only the broken upper part should be removed with the remainder of the fixture remaining in the bone. To completely remove a fractured implant, a trephine bur or surgical bur should be used to remove the surrounding bone. Severe bone defects often occur as a result after fixture removal. In Thomas et al. [1], the causes of implant fractures included defects in implant design or materials, improper placement of the prostheses, and overload. As also shown in previous studies, 59% of fractured implants exhibited loosening or abutment or screw fracture, marginal bone loss, and peri-implant inflammation before fracture [2]. Based on these findings, if precautions are taken by analyzing the phenomena that may occur before implant fracture, it may be possible to prevent implant fracture to some extent. The purpose of this study was to evaluate the factors that may affect implant fixture fractures.

## Methods

This retrospective clinical study was conducted after approval from the IRB (Institutional Review Board, IRB no. B-1910-568-112) of Seoul National University Bundang Hospital. From 2007 to 2015, patients who had fractured implant fixtures removed at the Seoul National University Bundang Hospital were included in the study. Based on patients’ medical records and radiographs (panoramas and periapical radiographs), the following factors were investigated: sex, age, implant type, implant diameter and length, bone grafting material, parafunction, presence of mesiodistal cantilever (only in single crowns), crown/implant ratio (C/I ratio), types of upper prostheses, the time of implant fracture, clinical symptoms before implant fracture, treatment of the fractured implant, and complications. Primary and secondary stability of the implant that was replaced after removal of the fractured implant was measured with the ISQ (implant stability quotient) using Osstel mentor (Integration Diagnostics AB, Goteborg, Sweden).

### Mesiodistal cantilever

**Fig. 1:**
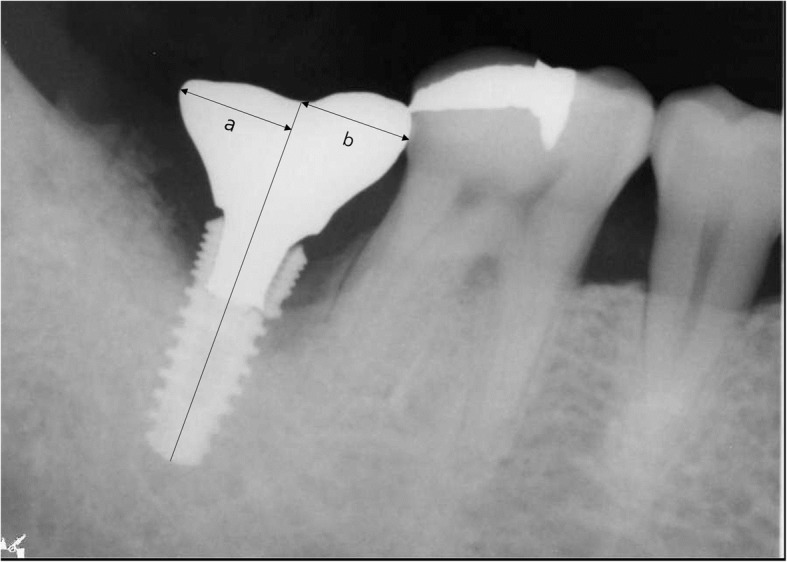
The method of determining a single implant cantilever (mesiodistal cantilever). If the difference between the length of “b” (distance from the midline to the longest part of the mesial) and the length of “a” (distance from the midline to the longest part of the distal) is greater than 2 mm, it is considered a mesiodistal cantilever. In this figure, fixture fracture occurred at the bottom of the prosthesis fixation screw and marginal bone loss were observed up to this site

The criteria for a mesiodistal cantilever for a single crown implant prosthesis are cited in a 2010 study by Kim et al. [3]. After the final prosthesis was installed, the presence of a mesiodistal cantilever in the single crown implant was confirmed using panoramic or periapical radiographs. The distance from the centerline parallel to the long axis of the implant to the height of contour of the prosthesis was measured. If the difference in distance between the mesial and distal part is more than 2 mm, it is considered a cantilever (Fig. 1).

### Crown/implant ratio

**Fig. 2:**
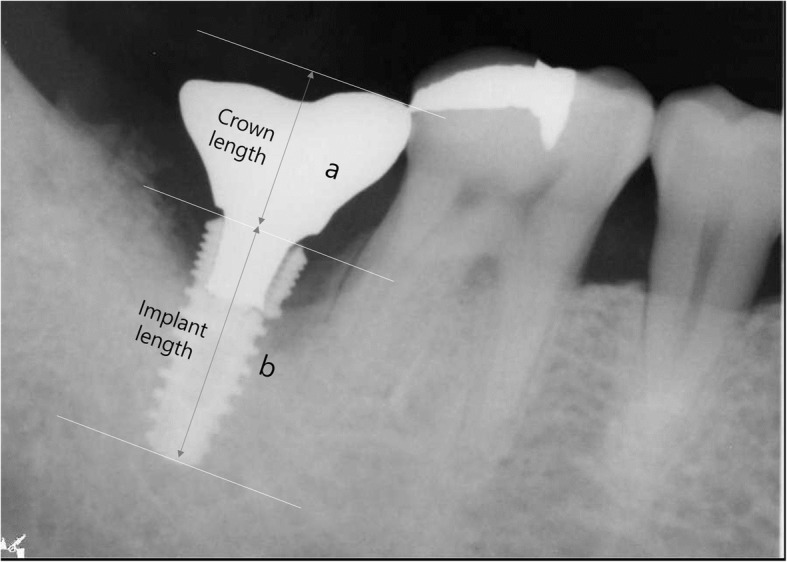
The method of measuring the crown/implant ratio

The crown/implant ratio (C/I ratio) in this study was measured using periapical radiographs. The length of crown “a” was measured as the distance from the implant platform to the highest point of the crown, and the length of implant “b” was measured from the implant platform to the implant apex. Each was measured perpendicularly to the implant platform. The crown/implant ratio was calculated by dividing the length of the crown by the length of the implant (Fig. 2).

### Remove the fractured fixtures

Implants with some degree of bone loss or mobility are removed by destroying osseointegration using forceps. However, for implants with little bone loss and strong osseointegration, the flap is elevated and the bone around the implant is removed using a thin surgical bur, etc., and then removed with forceps. Several companies provide implant removal kits. Using a trephine bur that fits the diameter of the implant, the implant can be removed by affecting only the bones around the implant. Kits provided by some companies destroy osseointegration in a unique way. After strongly connecting the kit to the implant hex driver, osseointegration is destroyed when the operator applies a strong reverse torque. Proper use of such kits minimizes trauma and permits implant removal.

## Results

A total of 13 implants were fractured in 12 patients (9 men and 3 women). Three of the 13 implants were premolars and the remaining 10 were molars. Six of the implants were broken in the maxilla and 7 in the mandible (Table 1). Patient age at the time of fracture ranged from 28 to 88 years with an average of 59.3 years. Seven of the 13 implants were placed at Seoul National University Bundang Hospital, while the other 6 were placed at a local clinic. The implant types placed at our institute were one Osseotite (Zimmer Biomet, IN, USA), one GS II (Osstem Implant Co., Busan, South Korea), one US III (Osstem Implant Co., Busan, South Korea), two GS III (Osstem Implant Co., Busan, South Korea), and two Implantiums (Dentium, Suwon, South Korea). The types of implants placed in the other dental clinics could not be clearly identified. The average duration of healing from implant placement to prosthetic completion was 5.9 months. The underlying disease present was hypertension in 3 patients, 2 of whom were taking antithrombotic drugs. In addition, one patient had a history of angina, one had dementia, one had hyperlipidemia, and one had undergone liver transplantation. There was one smoker among the 12 patients.

**Table 1:** Sex and location of the fractured implant

Patients			Total
Sex	Male	9	12
	Female	3	
Location	Maxilla	6	13
	Mandible	7	
	Premolar	3	13
	Molar	10	

The diameters and lengths of the implants are shown in Table 2. Five of the six implants that were not placed at our institute used an estimated value considering the enlargement of the radiographs. One implant could not be measured because the patient had a fractured fixture and a lost fractured segment.

**Table 2:** Diameter and length of the implants

Implants		*N*	Total
Diameter	3.8 mm	2	12
	4.0 mm	3	
	4.5 mm	5	
	5.0 mm	2	
Length	10 mm	1	12
	11.5 mm	4	
	12 mm	4	
	13 mm	3	
Unable to measure		1	13

The upper prosthesis consisted of 6 single implant crowns and 7 splinted prostheses. The mesiodistal cantilever of the single implant crown was measured. There was one mesiodistal cantilever in the study. The average crown/implant ratio for the 12 implants, except for one implant that could not be measured because the crown was removed at the time of visit, was 0.83:1. The clinical symptoms that were observed before fracture were screw loosening in 4 of the 7 implants placed at our institute. Repetitive screw loosening was observed in three of the four implants. Peri-implantitis was observed in 3 of the 7 implants and peri-implant mucositis was observed in one implant. Peri-implant bone resorption was observed in three of the six implants placed at the local clinic and screw loosening was observed in one implant. Periodontal issues were found in 6 of the 13 implants. Only one implant exhibited bleeding on probing (BOP). All of the fractured implants were removed and 12 of the 13 sites were re-implanted. Oral parafunction was observed in two patients: one with sleep bruxism, and one with attrition due to a strong chewing habit. The seven implants placed at our institute took an average of 61.7 months from prosthesis completion to implant fracture. None of the implants failed and were removed during the mean follow up period of 54.6 months.

## Discussion

Although the number of samples was small in this study, the fracture frequency was high in men. In addition, 10 of the 13 fractured implants were molars and the remaining 3 were premolars, suggesting that strong occlusal forces resulted in fracture of the implant. As noted in the work of Patterson et al. [4], a common cause of implant fracture is metal fatigue. When stress is applied intensively to a localized area, fractures tend to occur. According to Kenji et al. [5], most of the implant fractures occur in the premolar and molar regions. In the case of a single tooth restoration, most of the implant fractures occurred in posterior teeth. Rangert et al. [2] also reported that 90% of implant fractures occurred in posterior teeth. Occlusal forces are generally known to act most strongly in first molars. Eckert et al. [6] also reported that occlusal forces act strongly in the posterior region close to the masticatory muscle and jaw joints. Many forces act in different directions from the long axis of the implant. Therefore, fixture fractures can easily occur in the posterior region.

Sleep bruxism or clenching has also been reported to affect implant fracture. Chrcanovic et al. [7] reported that implant diameter, length, presence of a cantilever, and sleep bruxism have a significant effect on implant fracture. Stoichkov et al. [8] also suggested that the cause of implant fractures may be due to inadequate occlusion or excessive bite force due to sleep bruxism and is more common in single crown implants than implants with fixed connected prostheses. Sleep bruxism habits have been reported to adversely affect the survival and success rate of implants and may be a major cause of implant fractures. Therefore, it is advisable to identify the presence of oral parafunction and take appropriate preventive measures before implant treatment [9].

However, to accurately diagnose sleep bruxism and clenching, polysomnography is regarded as the gold standard, but has the disadvantage of being costly and time-consuming. Presently, there is no clinically complete method for diagnosing oral parafunction. Therefore, it is necessary to check not only paperweights, but also for hypertrophy of the masseter muscles, teeth attrition, and excessive oral tori. If deemed necessary, it may be a good idea to do perform a thorough sleep bruxism examination. If the test indicates that sleep bruxism is present, a stabilization splint, or botulinum toxin injection may be necessary as a preventive measure to reduce the occurrence of complications by lowering the symptoms of sleep bruxism and excessive bite force applied to the teeth [10–14].

In this study, generalized teeth attrition was identified in two male patients due to severe sleep bruxism and strong chewing habits. However, this study is a retrospective observational study with limitations that can only be judged by the results recorded in medical records. In addition, it is possible that more patients had oral parafunctional habits because the patients who exhibited them were not well diagnosed.

The correlation between the implant diameter and fracture of the implant fixture has been reported to be low [15–18]. In this study, the number of cases was insufficient, but the diameter of the fractured implants varied in 2 cases by 3.8 mm, 3 cases by 4 mm, 5 cases by 4.5 mm, and 2 cases by 5 mm. However, it should be noted that many papers report that the smaller the diameter of the implant, the greater the possibility that fracture occurs [7, 19–21].

The correlation between implant length and implant fixture fracture has been reported to be high. In particular, Chrcanovic et al. [7] reported that the probability of implant fixture fracture increases by 22.3% as the length of the implant increases by 1 mm. Most of the fractured implants in this study were implants with a length greater than 11.5 mm. However, a study by Tabrizi et al. [18] and Lee et al. [21] reported no significant correlation between implant length and fixture fracture. Therefore, further research is necessary in this area.

Papers with conflicting views on the relationship between sex and implant fracture have been published. Gargallo Albiol et al. [20] reported more fractures in men. However, Tabrizi et al. [18] reported that sex had no significant effect on implant fracture. In this study, 9 of the 12 total patients were male. The reason for more implant fractures in men may be due to the higher occlusal forces of men compared to women. Fixture fracture distribution according to age ranged from 28 to 88 years in this study, but all patients except for one who was 28 years old were over 50 years of age. In general, the reason why the age of the patients is high is that the age at which teeth are lost is higher in older people compared to younger people. Therefore, the percentage of young people in the implant treatment group was low.

After the implantation was completed at our institute and the upper prosthesis was completed, all of the implants that fractured during the follow-up period were found to have clinical symptoms such as screw loosening, bone loss, and peri-implant inflammation. In addition, bone resorption was observed in all the implants that had been implanted at the other clinic at the initial visit except for one implant that had already fractured. In particular, patients at our institute who had two implants fractured at the same time had splinted prostheses after two internal connection type implants were installed in the #15–16 area. Because of sleep bruxism, stabilization splints were made with hard resin. However, the device was not used well because the patient felt inconvenience. So, repetitive screw loosening was observed at 42 and 48 months after implant installation, and the patient reported feeling hypo-occlusion after feelings of discomfort at the site at 59 months after implant installation. Fixture fracture in both implants was confirmed.

Kim et al. [22] reported a case of implant failure in which the fracture occurred at the third thread. In a paper by Morgan et al. [23], fracture could easily occur if the peri-implant bone resorption progressed to the third thread, the weakest part of the implant fixture. Koller et al. [24] and Hsu et al. [25] found that marginal bone loss appeared prior to implant fracture, which may have an effect on implant fracture. As a result, when an overload is first applied to the implant, bone resorption occurs around the implant, and fracture may easily occur if the bone resorption progresses to the fracture weakened site. In addition, according to Tabrizi et al. [18], the loosening of the screw occurs before implant fracture as a precursor symptom.

Of the 7 implants that were installed at our institute and fractured, 6 were internal connection types and 1 was an external connection type. The number of cases was insufficient to obtain statistically significant results, and few existing studies have supported this finding. However, the reason why screw fracture occurs more frequently in the external connection type and abutment fracture or fixture fracture more frequently in the internal connection type was explained by Yi et al. [26] in 2018. When a force is applied to an implant, the internal connection type and the external connection type show structurally different force distributions. When a force is applied to the external type implant, the stress is first transferred to the screw, and the screw is broken first before the screw delivers enough force to break the implant fixture. However, in the internal type, there is a mechanical interface between the abutment and the fixture, and when stress is applied, it is transferred to the abutment and fixture, causing fixture failure.

## Conclusion

Care should be taken during implant installation or prosthesis placement to avoid implant fracture. Loosening of the screw, peri-implantitis, and loss of marginal bone are observed before implant fixture fracture. If a symptom is found during implant use, care should be taken to prevent its fracture.

## Data Availability

All data analyzed during this study are included in this published article.

## Abbreviations

ISQ: Implant Stability Quotient
C/I ratio: Crown/implant ratio
BOP: Bleeding on probing
